# ‘Change4Life Smart Swaps’: quasi-experimental evaluation of a natural experiment

**DOI:** 10.1017/S1368980016000513

**Published:** 2016-03-22

**Authors:** Wendy L Wrieden, Louis B Levy

**Affiliations:** 1 Human Nutrition Research Centre, Institute of Health and Society, Newcastle University, Room M1.151, William Leech Building, Medical School, Framlington Place, Newcastle-upon-Tyne NE2 4HH, UK; 2 Public Health England, London, UK

**Keywords:** ‘Change4Life’, Lower-fat dairy, Lower-sugar drinks, Plain breakfast cereal

## Abstract

**Objective:**

To evaluate the impact on food purchasing behaviour of the ‘Change4Life Smart Swaps’ campaign to encourage families to make small changes to lower-fat or lower-sugar versions of commonly eaten foods and drinks.

**Design:**

Quasi-experimental study comparing the proportion of swaps made by an intervention group (267 families who had signed up to the ‘Smart Swaps’ campaign promoted through various media, including television and radio advertising in early 2014) and a comparison group (135 families resident in Wales, signed up for ‘Change4Life’ materials, but not directly exposed to the ‘Smart Swaps’ campaign). During weeks 1, 2 and 3 of the campaign participants were asked to record their purchases of dairy products, carbonated drinks and breakfast cereals, using a mobile phone app questionnaire, when making a purchase within the category.

**Setting:**

England and Wales.

**Subjects:**

Families registered with ‘Change4Life’.

**Results:**

In weeks 2 and 3 a significantly higher percentage of the intervention group had made ‘smart swaps’ than the comparison group. After week 3, 58 % of participants had swapped to a lower-fat dairy product compared with 26 % of the comparison group (*P*<0·001), 32 % of the intervention group had purchased a lower-sugar drink compared with 19 % of the comparison group (*P*=0·01), and 24 % had made a change to a lower-sugar cereal compared with 12 % of the comparison group (*P*=0·009).

**Conclusions:**

In the short term a national campaign to change purchase habits towards healthier products may have some merit but the sustainability of change requires further investigation.

The prevalence of overweight and obesity has been increasing in the UK and worldwide since the 1980s and has now reached over 60 % of the UK adult population, with 30 % of those aged 2–15 years being classified as having excess weight^(^ ^1^ ^)^. It is well established that overweight and obesity increase the risk of chronic disease and thus impact on health-service budgets^(^ ^2^ ^)^. Findings from the National Diet and Nutrition Survey Rolling Programme (2008/09 to 2011/12) showed that people in the UK continue to eat too much salt, saturated fat and sugar, with intakes exceeding guideline amounts in both adults and children^(^ ^3^ ^)^.

‘Change4Life’, the social marketing component of the UK government’s strategy to halt the rise in obesity, was launched in 2009. One of the first campaigns was ‘How are the Kids’ where personalised feedback was provided to families who completed a brief questionnaire about their children’s eating behaviour and activity. Using a cluster randomised trial design Crocker and co-workers^(^ ^4^ ^)^ found little impact on attitudes and behaviour but an increased awareness of the campaign, which subsequent initiatives were able to build on. The ‘Change4Life’ commercial was rated the most motivational of twenty-nine different television commercials (from the UK, USA and Australia) by a representative sample of 1000 adults^(^ ^5^ ^)^. One of the key elements of this success appeared to be the absence of the use of the word ‘obesity’ with the focus on making healthy behavioural changes. More recent campaigns have included behavioural change techniques such as goal setting, monitoring and feedback, which have an increasing evidence base for success in weight management^(^ ^6^ ^–^ ^8^ ^)^. In the USA the ‘America on the Move’ programme promoted a small-changes approach to prevent excessive weight gain^(^ ^9^ ^)^. This was shown to be effective in preventing weight gain in children randomised to an intervention where families were asked to make two small lifestyle changes, one in regard to diet (replace dietary sugar with a non-caloric sweetener) and the other concerning physical activity (walk an extra 2000 steps per day), and compared with a control^(^ ^10^ ^)^. The potential energy saving and changes in diet quality of making simple substitutions in the diet of French adults were demonstrated in a model where light (reduced-sugar or reduced-fat) versions were substituted for conventional foods^(^ ^11^ ^)^.

This approach was promoted in the ‘Change4Life’ campaign of 2014 (‘Smart Swaps’) in which families were encouraged to make small changes in their food purchases by substituting lower-fat versions of milk and cheese and reduced- or no-sugar versions of fizzy drinks and breakfast cereals^(^ ^12^ ^)^. These swaps were chosen as the ones most likely to have a positive effect in reducing fat, sugar and energy in the family diet. A novel feature of the evaluation of this campaign was the use of a mobile phone app to collect data.

The aim of the present work was to evaluate the impact of the media campaign ‘Change4Life Smart Swaps’ on the proportion of swaps made by exposed (intervention) *v*. non-exposed (comparison) families.

## Methods

### Participants

Data were obtained from families who had signed up to the ‘Change4Life Smart Swaps’ campaign. This campaign was promoted through various media, including television and radio advertising in early 2014 with a television advertisement broadcast on English television regions in January 2014. The comparison group consisted of families who had signed up for ‘Change4Life’ materials in the past but resided in Wales where the sign-up facility was unavailable.

### Intervention

Intervention families received a ‘Smart Swaps’ sign-up pack providing information on the major contributors to fat and sugar in the diet along with suggestions on simple swaps that would reduce the content of these nutrients in the diet. Suggestions were chosen to reflect the top five contributors to fat and sugar in the diet of children assessed from the National Diet and Nutrition Survey^(^ ^13^ ^)^. Comparison families received no additional information.

### Data collection

Data were collected using a mobile phone app developed by TNS BMRC, a social research agency commissioned by Public Health England. During the first week (week 1) of the study, participants were asked specific questions about their purchases of dairy products, carbonated drinks and breakfast cereals and were asked to record these each time they made a purchase within the category, including taking a photograph for validation. The questions asked were specific to the food categories and the week, i.e. ‘Did you deliberately choose to buy any lower-fat dairy products/lower-sugar fizzy drinks/lower-sugar cereals last week compared to what you normally have?’ After each of these three separate questions participants were asked ‘What were these? During week 1 they also received their ‘Smart Swaps’ pack. Participants completed similar surveys in weeks 2 and 3 of the campaign. Other questions were asked about the participants’ perception of whether their eating habits had improved, and in the final week a check was made on how many of the suggested swaps each participant had carried out and whether ‘Smart Swaps’ encouraged them to make other healthy changes.

### Analysis

The proportion of participants recording a change in behaviour in the intervention and comparison groups was compared using the *χ* ^2^ test or Fisher’s exact test (for 2×2 tables).

## Results

### Recruitment and response rates

A total of 416 participants (67 % intervention group) were recruited to complete the questionnaires. After the first and third weeks response rates exceeded 80 %, with slightly lower rates after week 2. There were no significant differences in the response rates for the intervention and comparison groups or in the proportions of households with children aged 11 years and under. However, for the overall sample and following weeks 1 and 3, there were slightly higher proportions of participants from higher social grades (A, B and C1) in the intervention group compared with the comparison group (Table 1).

**Table 1:** Response rates and sociodemographic details of the sample, ‘Change4Life Smart Swaps’ campaign, England and Wales, 2014

	Recruited		Higher social grade (A, B, C1)		With children aged 11 years and under	
Group	*n*	%	*n*	%	*n*	%
Recruited	416	100	281	68	268	65
Intervention	277	67	199*	72	181	65
Comparison	139	33	82	59	88	63
Week 1	357	86	245	70	233	65
Intervention	236	85	171*	74	157	67
Comparison	121	87	74	63	76	63
Week 2	318	76	215	69	195	61
Intervention	215	78	152	72	131	61
Comparison	103	74	63	63	64	62
Week 3	342	82	238	72	217	64
Intervention	229	83	171**	76	145	63
Comparison	113	81	67	62	72	64

Due to 2·5 % non-response for the question used to determine social grade, the percentage given is of the total who provided social grade information and will not equate to an exact percentage of the total sample who completed the questions at the particular time point. Significant difference between intervention and comparison groups: *P<0·05, **P<0·01.

### Self-reported diet and purchase information

In week 1 of the campaign approximately one-third of participants in both groups claimed that their diet was healthier than normal. However, in subsequent weeks there was a significant difference between the intervention and comparison groups, with almost half (48 % *v*. 28 %) and two-thirds (63 % *v*. 39 %) of those exposed to the campaign in weeks 2 (*P*<0·01) and 3 (*P*<0·001), respectively, claiming their eating had improved.

**Fig. 1:**
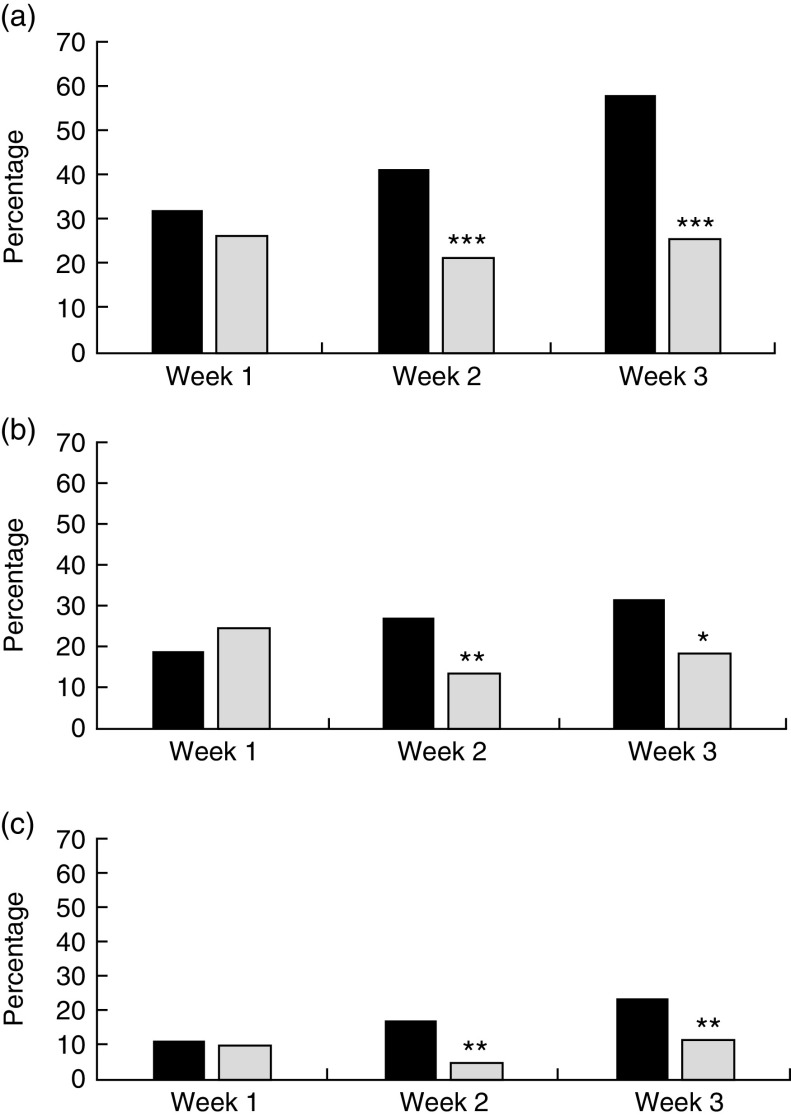
Changes in purchase behaviour following the ‘Change4Life Smart Swaps’ campaign: percentage of intervention group families (*n* 267; 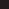) and comparison group families (*n* 135; 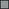) purchasing lower-fat dairy products (a), lower-sugar drinks or alternatives (b) and lower-sugar cereals (c) according to campaign week, England and Wales, 2014. Significant difference between intervention and comparison groups: *P<0·05, **P<0·01, ***P<0·001

There were no differences between the intervention and comparison groups’ purchases in week 1. In subsequent weeks the proportion of the intervention group reporting having made the swaps was significantly greater than in the comparison group. In week 2, 41 % (89/215) of the intervention group had purchased a lower-fat dairy product compared with 21 % (22/103) of the comparison group; and in week 3, 58 % (133/229) of the intervention group had made this swap but only 26 % (29/113) of the comparison group (Fig. 1(a)). Participants purchasing lower-sugar drinks constituted 27 % (59/215) of the intervention group and 14 % (14/103) of the comparison group in week 2; and 32 % (73/229) of the intervention group and 19 % (21/113) of the comparison group in week 3 (Fig. 1(b)). For lower-sugar cereals, the respective figures were 17 % (37/215) and 5 % (5/103) in week 2, and 24 % (54/229) and 12 % (13/113) in week 3 (Fig. 1(c)).

For both groups combined making changes in week 3, the most popular lower-fat dairy items purchased were lower-fat cheeses (41 %) and milks (32 %). However fifteen (eleven in the intervention group) participants claimed a swap to an item (e.g. diet cola or cereal) that was neither a lower-fat dairy item nor a spread when asked which lower-fat dairy products they had purchased. For lower-sugar drinks, 54 % of those claiming a swap in week 3 specifically stated a change to a lower-sugar cola drink, with a smaller number of others (15 %) changing to water (tonic, flavoured and tap). For lower-sugar cereal the most popular purchases were porridge or Weetabix (a wholewheat ready-to-eat cereal with approximately 4 % sugar; both 18 % of swap claimants in week 3).

**Fig. 2:**
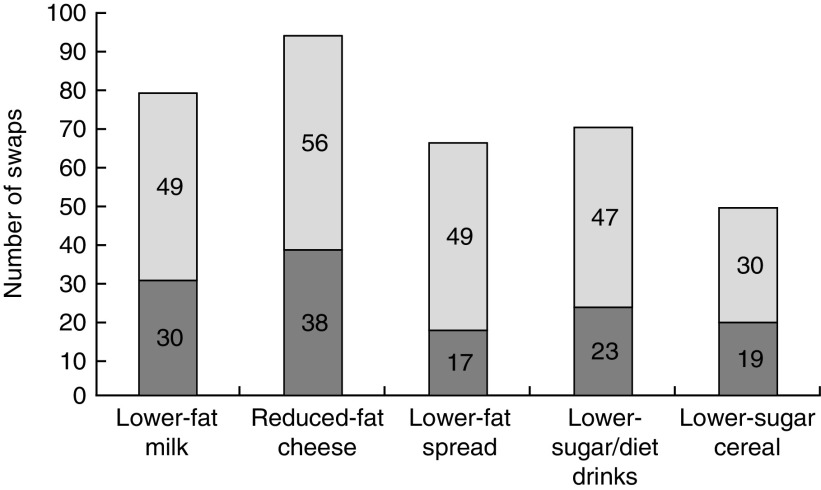
Number of swaps (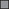, only swap; 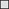, with others) claimed by week 3 in the intervention (*n* 267) and comparison groups (*n* 135) combined, ‘Change4Life Smart Swaps’ campaign, England and Wales, 2014

At the end of week 3, 74 % (169/229) of the intervention group and 30 % (34/113) of the comparison group (*P*<0·001) claimed they had tried a swap suggested by the ‘Smart Swaps’ campaign; multiple swaps were tried by some (Fig. 2).

In terms of other healthier changes made (and encouraged in the campaign pack), twenty participants said they were eating more fruit and/or vegetables, fifteen had changed their cooking practice and twelve claimed increased exercise/walking.

Further analysis of week 3 data showed that over half of the comparison group had heard of ‘Smart Swaps’ and eighteen had actually signed up.

## Discussion

It is known to be difficult to obtain evidence to demonstrate the impact of population-based public health campaigns. This is because robust evaluation designs such as randomised controlled trials are not possible due to the very nature of such campaigns^(^ ^14^ ^)^. The present study shows a potential method for small-scale evaluation of such campaigns by utilising a mobile phone app and recruiting a group as a control in a neighbouring country not exposed to the campaign. However, there was a small level of contamination of the comparison group, suggesting that some of those in Wales had been able to sign up.

That more than 400 000 families signed up for ‘Change4Life Smart Swaps’ in 2014 highlights the potential impact of such campaigns if the types of outcomes highlighted in the current evaluation were demonstrable across the wider campaign. Although not demonstrable in terms of cause and effect, it is interesting to note that data from the Kantar Worldpanel, a panel of 27 000 households in England, showed a 8·6 % reduction in the purchase of sugary carbonated drinks in January 2014 compared with January the previous year^(^ ^15^ ^)^.

A key strength of the current evaluation is the inclusion of a non-exposed comparison group in a neighbouring country in order to limit contamination. Similarly, the use of a mobile phone app to aid collection and personal verification of self-reports is likely to have facilitated the high retention rate^(^ ^16^ ^)^ and enabled a significant change to be demonstrated in purchase habits over the short period of the campaign. Owing to the delivery of the intervention pack in week 1 it is not surprising that patterns of purchase at this time were not significantly different; whereas greater changes occurred in the subsequent weeks as families had time to plan their food and drink swaps. The intervention was developed to engage and support families to identify and choose the most relevant swap for their family’s lifestyle. While the specific aspects of the intervention and supporting materials were not assessed, this approach appears to have been successful.

Limitations within the study include the fact that participants in the comparison group were likely to be more interested and engaged in the campaign messages than the general population, having previously signed up for similar campaigns. Although the campaign was aimed at those in social grades C2, D and E, the sample selected had a higher proportion of participants from the higher social grades (A, B and C1) but this could be because such families are more likely to participate in the research element of the campaign. However, there were no differences in the proportion of families making swaps by social grade, so it was assumed that the observed differences were due to the intervention rather than the higher proportion of social grades A, B and C1 in the intervention group compared with the comparison group. The data were also self-reported and could be subject to social desirability bias^(^ ^17^ ^,^ ^18^ ^)^. Additionally, the mobile phone app was not formally verified against a gold standard reporting mechanism.

Overall, the present evaluation has shown that, in the short term, the ‘Change4Life Smart Swaps’ campaign positively affected food and drink choice in a sample of interested individuals. While this does not definitively demonstrate the impact of the campaign, the small significant differences between the groups at weeks 2 and 3 demonstrate the potential impact of the campaign given the wide sign-up in England. The potential to build on the use of mobile phone apps to help record and verify food choices more easily, together with utilising increasing insight from behaviour change techniques and more focused messages within public health campaigns, continues to offer opportunities for further evaluation.
